# People’s experiences of distress and psychosocial care following a terrorist attack: interviews with survivors of the Manchester Arena bombing in 2017

**DOI:** 10.1192/bjo.2022.2

**Published:** 2022-02-03

**Authors:** John Stancombe, Richard Williams, John Drury, Hannah Collins, Lizzie Lagan, Alan Barrett, Paul French, Prathiba Chitsabesan

**Affiliations:** Young People's Mental Health Research Unit, Pennine Care NHS Foundation Trust, UK; Welsh Institute for Health and Social Care, University of South Wales, UK; School of Psychology, University of Sussex, UK; Complex Trauma and Resilience Research Unit, Greater Manchester Mental Health NHS Trust, UK; Oldham Healthy Young Minds, UK; Manchester Resilience Hub, Pennine Care NHS Foundation Trust, UK; and School of Health Sciences, University of Salford, UK; Research and Innovation Department, Pennine Care NHS Foundation Trust, UK; and Faculty of Health, Psychology and Social Care, Manchester Metropolitan University, UK; Young People's Mental Health Research Unit, Pennine Care NHS Foundation Trust, UK; and Faculty of Health, Psychology and Social Care, Manchester Metropolitan University, UK

**Keywords:** Distress, major incidents, psychosocial care, secondary stressors, service responses

## Abstract

**Background:**

Distress after major incidents is widespread among survivors. The great majority do not meet the criteria for mental health disorders and rely on psychosocial care provided by their informal networks and official response services. There is a need to better understand their experiences of distress and psychosocial care needs.

**Aims:**

The aims of our study were to enhance understanding of the experience of distress among people present at the Manchester Arena bombing in 2017, identify their experiences of psychosocial care after the incident and learn how to better deliver and target effective psychosocial care following major incidents.

**Method:**

We conducted a thematic analysis of semi-structured interviews with 18 physically non-injured survivors of the Manchester Arena attack, who registered with the NHS Manchester Resilience Hub.

**Results:**

Distress was ubiquitous, with long-lasting health and social consequences. Initial reluctance to seek help from services was also common. Early and open access to authoritative sources of information and emotional support, and organised events for survivors, were viewed as helpful interventions. Inappropriate forms of psychosocial and mental healthcare were common and potent stressors that affected coping and recovery.

**Conclusions:**

This paper extends our understanding of how people react to major events. Provision for the large group of people who are distressed and require psychosocial care may be inadequate after many incidents. There is a substantial agenda for developing awareness of people's needs for psychosocial interventions, and training practitioners to deliver them. The findings have substantial implications for policy and service design.

## The psychosocial and mental health effects of major incidents on survivors

Distress after emergencies is very common, with adverse psychosocial consequences and functional impairment for many who may never meet the criteria for mental health disorder.^1–3^ However, a recent international review concluded that we understand little about the course of psychosocial distress after major incidents.^4^ Understanding how people react and behave and their psychosocial and mental health needs before, during and after major incidents is crucial to planning and delivering responses.

The psychosocial effects of extreme events can be influenced by a complicated combination of primary and secondary stressors.^5^ Primary stressors arise directly from the event, such as being injured or fearing for one's life. Secondary stressors do not inherently have their base in the event, but in prior life events and societal responses to the disaster, such as personal or financial losses, and may be amenable to action to reduce their effects. Recent research has resulted in a growing awareness of the importance of secondary stressors and their potential to exacerbate and lengthen people's experiences of distress following major events.^1,6–8^ However, there is a dearth of research identifying which secondary stressors are particularly associated with mass terrorist events, and how they can be targeted through effective, timely psychosocial interventions.

Research on the psychosocial and mental health effects of terrorist attacks has been predominantly quantitative, and focused on identifying specific psychopathology, usually post-traumatic stress disorder and depression, within large cohorts of survivors. These studies privilege the prevalence of signs and symptoms and neglect survivors’ personal experiences, in terms of their experiences of distress over time and its impact on their everyday lives. Recently, research has demonstrated that qualitative approaches can provide valuable insights into people's experience of psychosocial distress and its course, which is lacking in quantitative approaches.^9–11^

## Principles and models of psychosocial care and mental healthcare

Although most people do not require access to services that deliver specialist mental healthcare after major incidents, the majority of people are likely to benefit from lower level, but nonetheless important, psychosocial interventions provided by their families, friends, colleagues or statutory and non-statutory organisations.^2^ There is some evidence indicating that people who are distressed may not develop disorders if they are offered sufficient support in a timely manner.^1^

Williams and Kemp have created what they call ‘the psychosocial approach’.^12,13^ Informed by Patel,^14^ it distinguishes people who are distressed from those who require biomedical interventions (based on trajectories of people's stress levels and dysfunction), and advocates aiding the greater number of distressed people through lower-intensity psychosocial care. Psychosocial care sets out to bolster the recovery environment, ensure that affected people are able to sustain their social connectedness and provide social support (defined as interactions that provide people who are embedded in a web of relationships with actual assistance that they perceive to be caring and readily available in times of need).^15,16^ This approach is now embedded in guidance from NHS England and NHS Improvement.^17^

Although there is evidence of the effectiveness of specialist therapeutic interventions for disorders, there has been less knowledge regarding the optimal provision of psychosocial care for the large proportion of people who develop mental health needs but do not meet threshold criteria for disorders.^18,19^ This represents an important gap in the literature.

## Use of psychosocial care and mental healthcare after mass terrorist events

The literature on people's use and experience of psychosocial care and mental healthcare after mass traumatic events is scarce. Apart from a few recent notable exceptions, there have been very few studies that reflect survivors’ voices.^10,11,20,21^ Consequently, relatively little is known about survivors’ experiences of services. This is an important gap in the literature. It is essential to learn more about these experiences to identify the aspects of care that meets survivors’ needs and strengthen their preparedness for future incidents.^22^

Unmet healthcare needs after major incidents are associated with higher levels of psychosocial distress, post-traumatic stress, somatic symptoms and reduced social support. It is also unclear whether the unmet needs reported are because of a lack of healthcare or receiving unsatisfactory care.^22^ Unidentified barriers may prevent people from seeking or accepting care. People who develop distress in the wake of terrorist attacks may be reluctant to seek help, although the psychosocial factors underlying this restraint are not clear.^23^

## The Manchester Arena bombing: implementing psychosocial care and mental healthcare in practice

On 22 May 2017, a suicide bomber detonated an improvised explosive device in the foyer of the Manchester Arena, killing 23 and injuring 239 children and adults. Approximately 19 500 people were present at the Arena, and a high number of children and young people were involved.

The incident triggered an immediate response from the multi-agency partnership in Greater Manchester that established the NHS Manchester Resilience Hub, which facilitated an assertive outreach and screen model with both public health and clinical components.^24^ The Hub was operational 7 weeks after the incident.

The Hub invited concert attendees to complete online screening questionnaires on stress, anxiety, mood and functioning, using a customised web portal. This screening was repeated at 3-month intervals in the first year, then 6-month intervals and annually after 3 years. Respondents were assessed and allocated to psychosocial care pathways according to their level of need.

As we write, over 3500 people have used the services provided by the Hub. This cohort presented an opportunity to increase our understanding of their experiences and the course of distress among survivors following the incident at the Arena, as well as the part that social factors, such as family and personal relationships, support services and the wider community, have played in their coping and recovery.

## Objectives

The objectives of the research reported here were: (a) to enhance understanding of the development, expression, mediation and mitigation of distress among people following the Manchester Arena event; (b) to identify what experiences of psychosocial care after the incident have helped or hindered people in their coping and recovery; and (c) to use participants’ experiences to learn how to better deliver and target effective psychosocial care following major incidents in future.

The whole study is reported in this paper and another. There are cogent reasons for separating the two papers. First, it was not practical to combine them given the breadth and depth of each paper. Second, they cover different dimensions of the psychosocial response to major incidents. In this paper, we explore survivors’ distress experiences and opinions about the state-funded support services. The other paper explores the informal support afforded by families, friends and wider social relationships.

## Method

### Outline

The study used cross-sectional design and qualitative research methods, employing semi-structured interviews to identify the experiences of people who registered with the Hub in Greater Manchester and their opinions about what interventions assisted them or otherwise. The full interview schedule is in the Supplementary Material available at https://doi.org/10.1192/bjo.2022.2. The interview was organised around the following topic areas: the social context before the event (e.g. ‘How would you describe what life was like for you before the Arena event?’); experiences at the event and immediately afterward (e.g. ‘Going in to as much detail as you feel comfortable with, what did you experience at the Arena that night?’); and social influences on coping and recovery (e.g. ‘Looking back, who or what has helped you cope or recover from the event?’ ‘Is there anyone or anything that has hindered you in your coping and recovery?’).

The interviews were conducted with targeted groups of adult participants (*n* = 18, split across three subgroups) who showed personal responses to the routine screening measures adopted by the Hub that were consistent with one of the three broad patterns (‘mild’, ‘moderate’ and ‘severe’ responses) in which people respond to emergencies and disasters.^25–27^

### Constructing the sample for the qualitative research

All Hub registrants were invited to indicate whether they wished to participate in future research, and this created a subset of registrants from which the sample was drawn. A purposive diversity sample (*n* = 18) was constructed from people in this subset who met the eligibility criteria (*n* = 262), on the basis of the scores of eligible persons on the Manchester Hub Screening measures, which included the Trauma Screening Questionnaire (TSQ),^28^ the Patient Health Questionnaire-9 (PHQ-9),^29^ the Generalised Anxiety Disorder-7 (GAD 7)^30^ and the Work and Social Adjustment Scale (WSAS).^31^ The measures are standardised and validated, with established clinical cut-off points.

Eligible people were defined as those who were directly affected by the Arena event (each participant had attended the concert at the Arena in May 2017, but none had been physically injured by the bomb), had at least one assessment on the Hub's psychometric screening measures at the 3- and 6-month post-event time points, and were aged ≥18 years on the date of their initial assessment.

There were no direct refusals to take part in the study from any of the Hub registrants who were invited to participate. However, some people were unable to respond within the time frame of the study.

The researchers endeavoured to ensure the sample contained people with a spread of age (18–24 years: *n* = 9, 25–44 years: *n* = 3, 45–55 years *n* = 6, mean average: 33.4 years), home addresses inside (*n* = 4) and outside (*n* = 14) Greater Manchester, and varying degrees of exposure to the incident; and that parents (*n* = 9) and young people (*n* = 9) were represented.

An important criterion for choosing the sample size was to ensure that our cohort included people with a range of responses to the incident. Our perception, following rigorous analysis of the interview transcripts, is that we achieved thematic saturation within each of the three distress subgroups with the final sample size of 18 participants (mild: *n* = 7, moderate: *n* = 6, severe: *n* = 5).

### Definitions

One of the challenges in people's experiences of major incidents is agreeing definitions of the terminology used. One term that requires greater clarity is ‘distress’. There are two broad approaches to defining it. First, some of the literature refers to distress being composed of symptoms of anxiety, depression or post-traumatic stress disorder.^32^ At the outset, we used the results of the screening measures to define in this way the levels of distress experienced by three groups of participants. Three subgroups were defined for this study: mild, moderate and severe response. People in the mild response subgroup had initial screening scores as follows: TSQ score <6, PHQ-9 score 0–9, GAD-7 score 5–9 and WSAS score 1–10. People in the moderate response subgroup had initial screening scores as follows: TSQ score 6; and/or PHQ-9 score of 10–19 or a score of 1 on the PHQ-9 self-harm item; and/or GAD-7 score 10–14 and/or WSAS score 11–20. People in the severe response subgroup had initial screening scores as follows: TSQ score ≥6 and/or PHQ-9 score 20–27 or a score of ≥2 on the PHQ-9 self-harm item; and/or GAD-7 score ≥15; and/or WSAS score ≥21.

The second common use of distress is in relationship to emergencies to depict people who have a range of experiences that are anticipated, and usually much broader than symptoms of common mental disorders. Other accounts organise these potential experiences into emotional, cognitive, social and physical domains.^33^ We report our judgements of our participants’ experiences by examining the transcripts of 18 interviews, and report the frequencies of these experiences reported by participants compared with reports in the disaster literature. This research allows us to offer a third approach to defining distress on the basis of the experiences of people who say that they have been or are distressed.

### Conducting the qualitative interviews

Each of the 18 participants undertook a single semi-structured interview conducted by one of two researchers (H.C. and L.L.) by telephone. The interviews were conducted over a 4-month period, between October 2019 and January 2020. Participants were asked about their experiences at the time of the bombing and in the intervening period. Each interview lasted up to 1 h and focused on their experiences and interpersonal factors that appeared to them to have helped or hindered their recovery. Each participant was enabled to have a person of their choice present to support them and was offered follow-up support. No-one asked for a supporter to join them, and one person took up the offer of support. Each interview was recorded with the permission of the participant and was transcribed verbatim. Each transcript was read by three researchers (J.S., R.W. and J.D.) and the interviewers (H.C. and L.L.) and broad themes were identified. Each transcript was then subjected to detailed thematic analysis by three researchers (J.S., R.W. and J.D.), to identify important and common psychosocial themes. The emergent themes were mapped.

### Consent

Each participant gave consent in writing to take part in the interview, our recording their interview and its verbatim transcription, and our use of the anonymised data in this research.

### Ethical approval

Ethical approval was provided by the UK's Integrated Research Application System process (application number 255819).

### Analysis

The approach to thematic analysis was theory-driven and inductive. In this paper, we focus on participants’ accounts of the psychosocial care provided by the support services. We set out to identify experiences of importance to participants, within the broad question of support after the incident, but which were not known to us *a priori*. Each transcript was read by five of the authors including the two interviewers, and the issues that seemed important to the interviewees were coded (e.g. the variety of experiences of support). Each transcript was then subjected to detailed thematic analysis by J.D., J.S. and R.W.^34^ They independently coded and developed suggestions on themes before coming together to compare their definitions, and merge and split themes as appropriate. They agreed a thematic structure through this iterative process, which was tabulated with definitions and examples, for easy visual inspection, and its reliability was further checked by asking someone from outside the project Thomas Redmond, Pennine Care NHS Foundation Trust, UK, who used it with a subsample of the data.

## Results

At interview, participants were asked about their experiences at the Arena in the immediate aftermath of the attack. This provided detailed information in relation to what they witnessed on the night and their immediate responses. Their experiences were categorised as low, medium or high exposure based on the following criteria:

people in the low-exposure subgroup experienced pandemonium and perceived threat to life; people in the medium-exposure subgroup experienced pandemonium, perceived threat to life and witnessed mild blood injuries; people in the high-exposure subgroup experienced pandemonium, perceived threat to life and witnessed serious blood injuries and dead and dying people.

The levels of exposure of the three response subgroups are presented in Table 1. Statistical analysis confirmed that there was no evidence that the three response groups differed significantly in their level of exposure (*P*= 0.884, two-tailed Fisher's exact test).

**Table 1:** Level of exposure of the mild, moderate and severe response groups

	Low exposure, *n*	Medium exposure, *n*	High exposure, *n*	Total, *N*
Mild response	3	2	2	7
Moderate response	3	1	2	6
Severe response	2	0	3	5
Total	8	3	7	18

The overarching thematic structure of the study is shown in Table 2. The results reported in this paper are organised around two domains: ‘experiences of distress’ and ‘experiences of psychosocial care’. The superordinate themes and themes are illustrated by verbatim exemplars from the transcripts of the interviews. ‘Mild’, ‘moderate’ and ‘severe’ denotes the extent of their initial reaction based on their 3- or 6-month screening scores.

**Table 2:** Thematic structure

Superordinate themes	Themes
Domain: distress	
Patterns of distress	Distress is universal
	Duration of distress
	Variety of distress
	Meaning of distress
Secondary stressors compounding distress	Relationships
	Work
	Stresses on parents
	Media responses
	Perceived neglect
	Public response
Domain: informal social support	
	Sharing emotional concerns
Constraints on seeking support	Internal
	External
Experiences of support	Social validation
	Emotional support
	Instrumental/material support
	Informational support
	Giving support to others
	Knowing support is available
Being part of peer-support groups	Shared experience as group identity
	Actions to create or maintain connection with group
	Lack of identification with others
Peer-support group dynamics	Too much focus on trauma
	New friendships
	New support
	Personal growth
Domain: psychosocial care services	
Reluctance to seek help	Should be dealing with this
	Needs of others greater
Access to care	Not knowing where to go
	Help not early enough
	Knowing support is available
	Encountering barriers
	Being enabled
Suitability of care	Primary care
	Authoritative validation
	Promoting social connection

### Domain 1: experiences of distress

#### Superordinate theme: the patterns of distress

##### Theme 1: distress is universal

A minority of the interviewees (five out of 18) showed elevated scores on the screening tools, indicating that they might have or might be developing a mental disorder. However, all interviewees were clearly distressed, in terms of being demonstrably affected by their experiences, based on their own accounts. The following extract illustrates how one of the interviewees with mild distress described their reaction to the event:

‘… for the first 6 months after I couldn't enjoy activities without feeling guilty … that people had died and got injured … I'd think a bomb was gonna go off like it's gonna happen right now … I started having a panic attack … At the time, I was like … freaking out. I was … physically shaking and I can't breathe and everything is really intense. Like every noise was so much louder than before’ Participant 18 (mild).

Another person, with mild distress on the Hub's screening tools, and not considered to require follow-up by the Hub, made a plea on behalf of everyone in distress who might not come to the early attention of support services through the Hub's outreach and screen programme:

‘I wrote a big thing back to them [the Hub] saying like even though I don't feel suicidal and all of the things you're asking … just because I don't tick all of the boxes on this and you're not worried about me, doesn't mean I'm not suffering … Because we were still traumatised just in a different way’ Participant 17 (mild).

Based on the interviewees’ accounts, there were no significant differences between the mild, moderate and severe groups in terms of their experiences of stress or distress before the event or at the time of exposure at the Arena.

##### Theme 2: the duration of distress

Interviewees’ accounts of the course of their distress were reflected by the changes in their screening scores over time. At 2 years, the scores of everyone with moderate initial scores now had scores indicative of mild distress. By contrast, the severe scores group was less likely to show signs of recovery based on their screening scores. A greater proportion of people with higher initial distress at first screening reported more enduring distress compared with those with milder distress.

All people in the mild group reported improvements in their initial distress at interview. However, some in this group (three out of seven) were still troubled by persistent fears of recurrence and anxiety about their safety in public places and impaired functioning:

‘… I think I kind of denied that it [life] has changed, but I know that it has … like I don't really like going out on my own anymore like I used to do … but I don't anymore’ Participant 2 (mild).

Most people (nine out of 11) in the moderate and severe distress groups reported some improvement in their distress over time. Those in the moderate group (six out of six) were more likely to report improvement than the severe group (three out of five). However, many (four out of six) in the moderate distress group were still experiencing some distress at the time of interview. This predominantly involved fear of recurrence, hypervigilance in public places and social avoidance. In addition, some people with moderate initial distress were resigned to enduring some form of long-term distress despite having received therapeutic intervention.

##### Theme 3: the variety of distress

People reported a wide range of features of distress after the Arena incident.^33^ The frequencies of these experiences are summarised in Table 3 under the headings of emotional, cognitive, social and physical reactions.

**Table 3:** The range and frequency of distress

Emotional reactions	Cognitive reactions
Fear of recurrence: 13/18	Hypervigilance: 13/18
Upset/tearfulness: 13/18	Detachment, dissociation or denial: 8/18
Guilt, shame and self-blame: 9/18	Intrusive thoughts: 7/18
Fear and anxiety: 8/18	Confusion or disorientation: 6/18
Persistent low mood: 7/18	Reduced confidence or self-esteem: 4/18
Feeling overwhelmed: 7/18	Impaired concentration: 2/18
Anger: 4/18	Impaired memory: 1/18
Helplessness and/or hopelessness: 0/18	
Shock: 0/18	
Social reactions	Physical reactions
Avoidance: 13/18	Hyperarousal/startle reactions: 8/18
Withdrawal: 13/18	Difficulty in performing everyday functions: 7/18
Irritability: 1/18	Somatic complaints: 4/18
Interpersonal conflict: 0/18	Insomnia: 4/18
Regression: 0/18	Reduced appetite: 3/18
	Reduced energy: 2/18
	Headaches: 0/18

Fear of recurrence and hypervigilance in social gatherings or public places were the most commonly reported forms of distress that invariably accompanied each other. This was a common and uniform pattern in all distress groups.

Avoidance (‘any behaviour or actions to prevent uncomfortable feelings/thoughts’) and social withdrawal (‘any actions taken to withdraw, isolate or disconnect with others’) were the most commonly reported social reactions after the event (13 out of 18 interviewees). Some social withdrawal was a consequence of active avoidance of large crowds and fears of recurrence. In most instances, social withdrawal was not associated with avoidance of reminders or fear of recurrence. Many interviewees (ten out of 18) simply reported that they preferred to stay at home after the event, and lost interest in being with other people and attending pre-event social activities:

‘… for a long time, I stopped socialising with people, because I found it really hard to relate to people. I still find it quite hard to make friends if that makes sense’ Participant 10 (severe).

Interestingly, the patterns of social withdrawal differed across the distress groups. In the mild group, it tended to be relatively short-lived, with no more than a few days or weeks. However, some people in the moderate group (three out of six), and all of the severe distress group, reported social withdrawal in the days and weeks immediately after the event that became more enduring. In some cases, social withdrawal was linked to long-term changes in lifestyle, friendships and social group memberships.

Regarding their emotional reactions, many interviewees described being ‘upset’ and/or ‘tearful’ (13 out of 18) after the event. And this form of acute distress was common in all subgroups. More persistent emotional reactions and low mood lasting for months or longer were only reported by people in the severe distress group (four out of five).

Physical reactions, except for hyperarousal, were more likely to be reported by people with higher levels of initial distress. For example, sleep difficulties, loss of appetite, somatic complaints and physical inability to perform everyday functions were reported by some people with moderate distress (three out of six) and many with severe distress (four out of five). In terms of long-term effects on physical health, one person with moderate distress reported that they had developed irritable bowel syndrome in the aftermath of the attack. And another person with severe distress stated that they had been diagnosed with ‘stress-related angina’ following the event.

Shame or guilt were common emotional responses in both the acute phase and the longer term (ten out of 18 of the interviewees). Only a minority of people in the mild group (two out of seven) reported this as a feature of their experience of distress. In contrast, shame and guilt were more likely to be reported by people in the moderate distress group (four out of six), and by everyone with severe distress. The most common reason interviewees gave for feeling shame or guilt related to the intensity and duration of their distress (seven out of 18). Many felt that they were weak or inadequate for experiencing distress at all:

‘… and a few weeks after, I started to feel really guilty that I was even affected at all because I hadn't been physically hurt or lost someone … I still think it's not OK for me to sit and wallow, because nothing happened to me' Participant 17 (mild).

Guilt was also a feature of the accounts given by some interviewees who were parents. They described the guilt they experienced for exposing their children to the attack, and their perceived inability to provide adequate support afterward. Another parent felt guilty about leaving with their daughter and neglecting injured people. One young person stayed to assist and experienced guilt about perceived deficiencies in the care they delivered. However, reports of ‘survivor guilt’ were very uncommon (one out of 18).

##### Theme 4: the meaning of distress

The ways people made sense of their distress and the meaning that they attributed to it are important in understanding their suffering. A number of participants, for example, were surprised by the intensity of their distress post-event, its duration and the impact it had on their everyday lives. Often, they reported their personal experiences of distress as atypical of them, and as a threat to their perceptions of agency and self-control. Other people were surprised by the severity of their distress compared with their perceptions of the extent of their exposure and the physical effects of the event. As they struggled to make sense of their feelings, they tended to make self-evaluations that were predominantly negative:

‘… it took me by surprised at how it made me feel and for how long … yeah because I think when it came to that November when I got some help, I just thought well why am I still feeling like this I wasn't hurt, I wasn't injured, I didn't see anything, so what on earth's wrong with you …?’ Participant 12 (moderate).

#### Superordinate theme: the secondary stressors compounding people's experience of distress

Many interviewees (12 out of 18) described events, policies and practices that were not inherently based in or consequential to the incident itself, but which became sources of substantial stress and compounded their experience of distress arising from the event. These secondary stressors tended to be reported more frequently by people with higher initial distress and more enduring reactions (mild: 0.4 reports per interviewee; moderate: 2.0; severe: 3.6).

The most common exacerbating stressors were the responses of services, friends and family; work settings; the media and wider society. The stress associated with the response of psychosocial and mental healthcare services was a recurring theme (seven out of 18), and related mainly to problems getting access to timely support or unhelpful experiences of care. We are reminded that, as well as being helpful, services, and the ways in which they respond to people who use them, may also be secondary stressors. This theme is highlighted later in this analysis.

##### Theme 5: relationship stressors

As reported in our other paper, partners, close friends and families were preferred for sharing personal experiences of distress and chosen sources of emotional support early on. However, people's negative perceptions of some people's responses to them inhibited further sharing and, in some cases (five out of 18), compounded their distress by affecting their coping and recovery:

‘… That made it [distress] worse … she wasn't a mum to me at that point, it was a good 6 months where she was my mum, but she wasn't being a mother … but at the time, she didn't see what was happening, like I did blame her a lot for a lot of it …’ Participant 13 (moderate).

##### Theme 6: work stressors

The majority of interviewees (14 out of 18) were employed at the time of the Arena event. Their experiences of the responses from people at work were mixed. Initial responses were predominantly positive; participants felt that work colleagues provided emotional support, and employers readily provided instrumental support in the form of reduced duties or paid leave. However, some (four out of 14) felt that this support was relatively short-lived and evaporated over time, as employers became impatient and their expectations of their employee's return to pre-event levels of performance increased. In some cases (three out of 14), people were forced to take long-term sick leave or resign from their jobs because of the stress caused by the lack of understanding and support from employers:

‘I had a really bad experience with work. … after about 6 months, I had to leave because I felt like I was being bullied … I was like the perfect employee … but, because of the PTSD [post-traumatic stress disorder], I was having panic attacks … It was kind of they lost any sort of empathy or patience with me … so they started saying things … like “you know it's convenient that you have panic attacks at work” and … “try living sort of 24/7 with this sort of thing”’ Participant 10 (severe).

##### Theme 7: stresses on parents

Many parents described the stress of identifying services for their children and their concerns about their children's recovery. They (five out of nine) reported that the distress they suffered that was directly associated with the event was compounded by stress arising from their roles and responsibilities as parents, which became secondary stressors:

‘Having to constantly be there to support [daughter] … it made things quite difficult, obviously as a single parent I do have to work … I did take unpaid leave … You're a parent … and I am also a nurse and then that kind of comes out as well. Now looking back on it, it has been really tough’ Participant 14 (mild).

##### Theme 8: the response of the media

Many interviewees (13 out of 18) reported that exposure to news and social media coverage of the event exacerbated their distress. Some interviewees (four out of 18) felt that the responses of news or social media were a direct and substantial source of stress that compounded their distress:

‘Facebook was very brutal and very nasty. Just people making comments that were not very understanding and putting really horrible stuff that kinda worsened me’ Participant 18 (mild).

##### Theme 9: perceived neglect – what about us?

There were also perceptions (five out of 18) that people's psychosocial experiences and mental health needs were viewed as less important than the needs of bereaved and physically injured people in wider society. This apparent inequity was both a source of personal stress and also affected their perception of entitlement to support:

‘I cannot tell you how negative [it] made me feel about myself when Theresa May and everybody was on the news saying, “our thoughts are with the bereaved and the injured people” and I'm sat at home, my life had just gone before me … and I'm thinking I shouldn't feel like this because I'm okay, I walked away, why do I feel like this, they've not mentioned us so we should be okay and then it went on and on and on and we never got mentioned …’ Participant 7 (severe).

##### Theme 10: the public responses of solidarity

The public response to the event through social and cultural acts of solidarity that were intended to demonstrate empathy and compassion, were sometimes experienced as a substantial source of added stress. Some interviewees (three out of 18) reported feeling disconnected from the public and cultural displays of support and this compounded their distress:

‘I don't think it was helping that I was having to go into Manchester every day and see all the “We love Manchester” signs … it was just getting worse and worse and worse, and I begrudged every single sign I saw, and I hated it and it wound me up you know … it really aggravated me’ Participant 8 (severe).

In other cases, interviewees experienced stress and disconnection from public displays of solidarity because they perceived them as inauthentic, media driven and incongruent with their everyday experiences of personal support.

#### Superordinate theme: reluctance to seek help from services

The interviewees turned to a wide range of support services for help with their distress (Table 4). However, an initial reluctance to seek help from these services was a common theme in our interviews (nine out of 18).

**Table 4:** Sources of support from services

Source	Number
Resilience Hub	16/18
Therapist	11/18
Counsellor	10/18
General practitioner	9/18
The Peace Foundation	3/18
Victim support	2/18

##### Theme 11: ‘I should be able to deal with this on my own’

Some people's reluctance to seek help from support services was based on negative self-evaluation of their ability to cope with distress. In some cases, reluctance was reported by people who were experiencing severe levels of distress:

‘I don't deserve to ring them (support service) … I should be strong enough to deal with all of this myself’ Participant 8 (severe).

In other cases, it was based on the interviewee's perception that the event was less stressful for them than for others and, therefore, their distress did not warrant support:

‘I feel like … like I know that I am lucky but then it makes me think like well some people did see a lot more and they need it [support] more than I need it, so like man up’ Participant 2 (mild).

The following extract demonstrates how self-denigration can result in some people dropping through the net of psychosocial care:

‘The [charity organisation] said somebody else will be in touch to take over, but they never got in touch … I should probably have picked up the phone and got in touch, …well, I won't do that because I'm just wasting people's time…’ Participant 12 (moderate).

##### Theme 12: ‘the needs of others are greater than mine’

Some people believed that care was only available to support people with greater needs. In some cases, they gave altruistic reasons for their reluctance to approach services:

‘I didn't necessarily ring them [the Hub] because I just thought there is probably other people who won't be getting the same level of support as me’ Participant 4 (mild).

Sometimes reluctance was based on the belief that support services were having to prioritise their response based on a hierarchy of needs. This made some feel that they were less important and unentitled to support:

‘In the sort of early days, where the only coverage was … the dead, the dead's families, the injured, you know and anyone else who was in the foyer and then everyone, witnesses, you sort of get put in this hierarchy and you don't feel like you're entitled to … the feelings that you're having’ Participant 10 (severe).

### Domain 2: experiences of psychosocial care services

Interviewees experienced a wide variety of psychosocial care from a range of services (see Table 4), and gave detailed accounts of how this ameliorated their distress and facilitated coping and recovery. However, interviewees also reported unhelpful experiences in relation to accessing psychosocial care and perceived shortcomings in what was provided by services.

#### Superordinate theme: the importance of early access to appropriate care

##### Theme 1: ‘not knowing where to go’

Many interviewees (ten out of 18) reported that they made active efforts to identify sources of formal support in the weeks after the attack, but did not know where they could obtain an appropriate service. Some reported relying on Internet searches to find appropriate help and support.

The majority of the interviewees (75%), as reflected in the people at the Arena and those supported by the Hub, were living outside Greater Manchester at the time of the event. Some perceived that services were centred in Manchester and were not accessible to them:

‘I felt annoyed as all the help and support seemed to be based in Manchester and I had nowhere to go … and I got pure rage and I sent over a really angry email to the woman who had written…saying that the language in it was all exclusive and…it excludes three-quarters of the country…’ Participant 1 (moderate).

##### Theme 2: early help – ‘it wasn't early enough’

Some interviewees (seven out of 18) thought that there could have been more immediate efforts to reach out to people who were affected by the event so that they did know where to turn if they were seeking psychosocial care. For example, some reported that the first outreach contact they received from the Hub, some months after the event, did not come ‘soon enough’:

‘I mean, I think the first screening … obviously we got the email from the Hub to say … I think the first one wasn't until quite a few months. I think 4 or 5 months after the attack … I think something more immediate would have been appropriate’ Participant 3 (mild).

Some (four out of seven) felt that the delays in accessing psychosocial care were not favourable to their coping and recovery:

‘I think if we would have got help sooner … the problem with having it 6 months down the line is you've started to recover a little bit for some things but then you have to go back and re-live it again, so it takes me backwards …’ Participant 12 (moderate).

Many parents (four out of nine) felt that it was difficult to get early help and advice about the best ways in which they could support their children. They described how this acted as a secondary stressor in that it compounded their own personal distress. Moreover, they felt that earlier access to appropriate psychosocial care might have enabled them to provide better support for their children and reduce their children's suffering:

‘I think for me particularly, and as a parent who was there, just to be able to know where to get help and support and advice from quicker than I did … because I did go out looking for it and I couldn't … I felt it might not have stopped the things that happened, but it would have given me a bit of reassurance that it was what we were doing the right thing to do’ Participant 14 (mild).

##### Theme 3: knowing that support is ‘out there’

Many people (eight out of 18) described how being aware that services were available and accessible, if needed, helped to mitigate their distress. Having information about whence they could turn if they decided to ask for help, gave reassurance. The following extract illustrates how this benefit was realised through retaining an information leaflet distributed by an official support service in Manchester in the days following the event:

‘…there were people handing leaflets out, so I took one and I kept that on my desk for a long time and just looking at it thinking I don't deserve to ring them …I shouldn't be ringing them, I should be strong enough to deal with all of this myself, but having it on my desk was a bit of reassurance, that if I ever felt like I needed it that I could call it… so maybe just having that information there was helpful in a way that I didn't realise at the time’ Participant 8 (severe).

Some interviewees (five out of 18), when describing the Hub, referred to the benefits of just knowing that they had a service available to them at the end of the telephone, if and when needed. Others thought that regular email contact from the Hub provided them with emotional support because it signified that their distress was not forgotten:

‘What I did appreciate was that I continued to get emails even though they were … sent to everyone it wasn't like they stopped after a certain amount of time … just to know that someone cares, and they know it has affected your life and always will’ Participant 17 (mild).

##### Theme 4: encountering barriers to accessing support

Many interviewees (ten out of 18) reported that it was unhelpful to repetitively encounter barriers when attempting to access care. Indeed, some (seven out of 18) thought that the stress of encountering service barriers compounded their experiences of distress, and thus acted as a secondary stressor. The barriers came in a variety of forms including, for example, being turned away for not meeting services’ threshold criteria for care and the delay of long waiting lists. There were several people (seven out of ten) who were experiencing severe distress early on who found long waits in accessing adult mental health service assessment unhelpful. A number of parents (three out of nine) reported how unhelpful it was to experience long waiting lists, ‘watchful waiting’ responses and what they perceived as inadequate interim support from child and adolescent mental health services when their children were experiencing acute distress:

‘… she was clearly showing signs of PTSD [post-traumatic stress disorder] quite early on … Anyway, they [child and adolescent mental health services] said we've got a waiting list, if you are concerned still in 8 weeks then call us back … and they sent out a trauma leaflet, which arrived second class 2 weeks later and it was addressed to [daughter] but it was an adult's trauma leaflet’ Participant 1 (moderate).

In one case, having encountered a series of ‘hand offs’ and ‘wrong doors’ in seeking suitable care, a young person described how the stress of struggling to find appropriate care deterred them from seeking official support and caused them to turn to self-medication:

‘… then it got to New Year, and it just went downhill from there. I was in contact with the Hub, and they were saying “try this place, this place and this place”… I don't know how many people I went to, and they were like “no”, so one particular place I went to … she was basically just very dismissive of it all … she pretty much said “you're not bad enough to treat right now, so we can't do anything for you” … that actually made me worse, and I didn't want to get any authoritative help after that because … I clearly can't be helped so what can I do? So, I then turned to alcohol, and I did take drugs’ Participant 13 (moderate).

The enhanced ‘outreach and screen’ model of the Hub was generally experienced as helpful by the interviewees. However, some who experienced the screening algorithm, with its inherent threshold criteria, as a barrier in accessing psychosocial support.

‘… instead of generalising everybody and sending them the same stuff and then if they don't tick the right boxes, then they won't get contacted or whatever. Maybe more a one-on-one …’ Participant 17 (mild).

##### Theme 5: being enabled to find the right help

Many people (nine out of 18) reported that it was helpful to have advice from the Hub, and other sources, in finding and accessing care when they did not know where to go or when they were encountering barriers. This sometimes involved signposting them to suitable or alternative psychosocial or mental healthcare services. In other cases, it involved fast-tracking or procuring psychosocial support and therapy services:

‘… we found it relatively quick getting therapy … and I'm very, very grateful to the Resilience Hub for bringing that forward like they did … but it's not happened for everybody unfortunately’ Participant 7 (severe).

#### Superordinate theme: care is available but is it the most appropriate care?

##### Theme 6: experiences of primary care

In the early aftermath, many interviewees (nine out of 18) turned to their general practitioners (GPs) when seeking assistance with their distress. They gave a variety of reasons for seeking support from primary care. The most common was suffering severe levels of distress that affected their everyday functioning. They also included: difficulty sharing distress with others; getting a sick note for work; concerns about impaired memory; and parents seeking advice on supporting their children. The majority (six out of nine) reported that the consultations with their GP were unhelpful. Many described encountering limited knowledge of the psychosocial effects of major incidents and appropriate support services, and inappropriate offers of care. The following extract highlights how one interviewee was apparently surprised and disappointed by the GP's response to their distress and the offer of care:

‘It was about what to expect … and he [GP] goes “right I'll print this leaflet off” and he said to me “you are pretty much ticking all of those boxes” and I went “I know; I know that …” it was almost like he was teaching himself as he was reading the letter’ Participant 1 (moderate).

Some were more openly critical of the care offered by their GP and did not feel that it was suitable for their psychosocial needs at the time:

‘I just knew that I had mild mood and anxiety … I just feel bad but not depressed … and I've got family that have got mental health issues so I … knew that I was different from them … I was going to the doctors [GP] and they were saying “take these pills you will feel better”, I'm saying “no I don't want to take those pills”, I want people to talk to me and help me to get through this rather than block it out. Participant 16 (moderate).

A number of parents (four out of nine) consulted their GP for advice in relation to how best to support their children. The majority (three out of four) stated that they were left disappointed by the care that was provided:

‘… the girls, I still kept them off school, they were just exhausted, and I phoned my GP, and I was like “so we have been at this, what do I do? …” and then my GP was like “I have absolutely no idea” he was like “I have never encountered this”… I didn't know where else to go for advice … so I did feel a little bit sort of helpless really because I didn't really know what the best things to do was really or what to do’ Participant 14 (mild).

##### Theme 7: the importance of authoritative validation and invalidation

A salient theme (ten out of 18 interviews) in relation to helpful psychosocial care was the experience of authoritative validation. Authoritative validation can be defined as the recognition or affirmation of a person's distress and entitlement to care by a person who is perceived to have specialist knowledge or expertise in relation to the psychosocial effects of major events. Authoritative validation both confers positive connotations to a person's distress and seeking support, and it challenges any negative self-evaluations, i.e. people seeing their distress or help-seeking as a sign of weakness or inadequacy.

In the first example, the interviewee describes how the first telephone contact with a professional at the Hub changed the way in which they viewed their distress and provided some relief:

‘… it [the Hub] was quite cathartic … I was really upset when I got off the phone but in a good way, because I could see that there was like a light at the end of the tunnel … and I know time's a healer and you have to wait certain things out … because I didn't know if I was going crazy, if it was a normal thing’ Participant 1 (moderate).

In other cases, authoritative validation involved making the distress understandable to a person, which draws on the science of how people react to and recover from traumatic events. However, examples of authoritative invalidation were also prevalent in accounts of experiences with services (four out of 18). These experiences can be powerful in exacerbating distress and act as secondary stressors:

‘I wanted someone neutral in terms of the therapist … where I could kind of say those things that I wouldn't necessarily say to my mum … that was when I started having CBT [cognitive–behavioural therapy], which at this point was … a massive step back to be honest … I'm here to talk about the fact that I have been part of a terrorist attack … she said to me “well you have 16 sessions from your doctor and if you were to abandon the sessions, then you might not necessarily get the care again in the future’ Participant 15 (moderate).

##### Theme 8: promoting social connectedness

This last theme concerns participants’ experiences of local support groups, family days and workshops that were organised by the Hub and other support services. Many interviewees (11 out of 18) reported benefitting from the opportunity to attend formal group events organised by services. Some people would have liked these events to have been provided sooner. Also, some people who did not have the opportunity to attend a formal group said they would have liked this form of support. Participants said these events were helpful because they were a means of making connections with other people who have shared experience who later became important sources of support in coping, adapting and recovery:

‘… there was two people I actually met from the [Hub] workshop last year, so I still keep in contact with them … so, yeah, I have met people through this experience, and … I can talk to them, and they can talk to me … not compare our experiences, but kind of help give each other ways to … cope’ Participant 11 (severe).

We do not develop this theme in detail here because our other paper covers informal support in more detail.

## Discussion

The qualitative interviews produced rich and detailed accounts of survivors’ experiences of distress over time, its impact on their everyday lives, and their perceptions of, and experiences with services.

A key finding is that distress during and after incidents is ubiquitous but not necessarily a function of psychopathology including mental illness. This supports a more dimensional and dynamic as opposed to a categorical view of distress, in that people differed in the degree of distress they experienced over time, rather than in kind.^35,36^ This draws attention to unresolved matters in the use of language. One of those is the way in which the term ‘distress’ is used. Based on the research reported here, we recommend use of the term as it is applied during and after emergencies to depict people who have a range of experiences that are anticipated, and usually much broader than symptoms of common mental disorders. Viewing distress as a group of symptoms of undiagnosed disorders is less helpful.

The common experiences that the distressed people we interviewed reported included feeling upset, fear, anxiety, fear of recurrence of the event, excessive vigilance at social gatherings and in public places, avoiding uncomfortable feelings and social withdrawal. Re-experiencing was less common, and anger and moral distress were rare. None of the interviewees spoke in terms that suggest they experienced shock and numbness. This suggests that the view of distress that is often used in mental healthcare and portrayed in public-facing leaflets may unintentionally reduce the chances of people's needs being recognised. It is important to recognise distress because the numbers of people affected are much greater than the numbers who screen positive for a possible mental disorder. This means that screening scores should not be used as a proxy for the potential need for services because there are likely to be people who are distressed but do not report severe levels of mental health symptoms, who are likely to wish to use services. Also, it may be necessary to adjust the lists of the common features of distress that are often included in information leaflets and digital resources.

In the introduction, we drew attention to recent reviews^4^ opining that relatively little is known about the spectrum and course of psychosocial distress apart from the broad categories or trajectories of response. The study reported here, on people's actual experiences, provides valuable insight into distress, which has not been the case with research more focused on identifying symptoms of psychopathology. Consistent with previous work on major events, the intensity of initial distress was strongly associated with enduring and debilitating distress.^37^ However, there were some reactions, which have drawn less attention in previous research, that appear to be associated with certain subgroups of distressed people. Physical reactions, for example, such as sleep difficulties, loss of appetite, somatic complaints and physical inability to perform everyday functions, were reported by people with higher levels of initial distress and more enduring functional impairment, as indicated by symptoms on screening tools. This association has also been reported elsewhere,^21,38^ and more recently in a longitudinal study after the terrorist attacks in Norway in 2011.^39^ We also found shame and guilt to be common emotions in the acute phase that were more often associated with longer duration of distress. Hence the results from this study underscore the body of literature, which indicates that shame has significant involvement in the intensity and course of distress following major incidents.^40^ Shame is likely linked to enduring distress through a web of bidirectional, psychosocial mechanisms.

We think that somatic reactions and shame might serve as early markers of the risk of more severe and enduring difficulties. It may be important to include them in enhanced psychosocial screening, and in targeting psychosocial interventions to mitigate the risk of distress becoming chronic. However, we acknowledge that these provisional findings, namely that certain types of distress may be specific to certain subgroups, need to be pursued in future grant-funded research.

From the interviews, we identified the importance of assisting people to overcome the worrying tendency of certain participants to isolate themselves in the short and longer terms. Social withdrawal, which was not associated with reminders of the event or fear of recurrence, was common early on, when it tended to be short lived. However, some people who were moderately or severely distressed reported early social withdrawal that became more enduring. In some of these cases, social withdrawal was linked to long-term changes in lifestyle, friendships and membership of social groups. Having functioning social networks is a key predictor of well-being and recovery from major incidents.^41,42^ Both short- and longer-term social withdrawal could limit access to particularly valued forms of support after events. Therefore, our view is that an early outreach programme is required to help people to avoid the risks of their withdrawing from social contacts.

The recovery trajectories and the enduring nature of distress found in our transcripts broadly fit with the existing literature.^43,44^ However, the screening data and our participants’ accounts suggest that the existing literature underestimates the number of people who take a long time to recover, and distress may be intense and persist over lengthy periods of time. This finding underscores the long-lasting health and social consequences of disasters. The implication is that planning should take account of these longer trajectories, not only for groups of people who suffer mental disorders, but also for people whose distress does not come to the attention of services.

In the beginning of this paper, we highlighted the important role that secondary stressors can play in compounding and maintaining distress after major events, and noted that our current understanding of the role of secondary stressors in the context of terrorist attacks is limited. Our findings advance our understanding; they elucidate the common and potent stressors that affected our interviewees’ coping and recovery. They show that inappropriate responses from their immediate friends and families in the form of social invalidation of their distress acted as an important secondary stressor. Inappropriate responses to their distress from their wider social context, such as those from employers, the media and the public, were also substantial secondary stressors. This finding suggests that, although social support is often considered protective, a more nuanced, multidimensional understanding of social support should recognise that inappropriate forms of social support are not only unhelpful, but may function as secondary stressors, potentiate distress and be detrimental to coping and recovery. However, we emphasise that inappropriate forms of psychosocial and mental healthcare can also function as secondary stressors; for example, authoritative invalidation and encountering barriers to accessing support services.

Our analysis has also highlighted that the predicaments of many parents in worrying about their children acted as secondary stressors. The difficulty associated with identifying appropriate psychosocial care combined with increased caring responsibilities when coping with their own personal distress, were a common experience.

The secondary stressors that emerged from the analysis were reported more frequently by people with higher initial and more enduring levels of distress. In our opinion, it is likely that there is a two-way rather than a linear relationship between distress levels and secondary stressors. Importantly, we argue that all of the stressors were consequential, socially mediated and preventable with timely and appropriate psychosocial care and intervention.

Previously, we cited previous studies that highlighted the association between unmet healthcare needs and higher distress, post-traumatic stress, somatic symptoms and social support. This paper advances our understanding of the relationship between unmet care needs and distress, which should be seen as important targets for more effective intervention in future.

First, our findings indicate that the ways in which people appraise their subjective experiences exacerbate their suffering and make them reluctant to approach formal caring services. We found that, as people struggled to make sense of their distress, they were often self-critical of their apparent inability to cope and recover. Many perceived this as a personal ‘weakness’, and felt that they should be ‘strong enough’ to manage their distress ‘on their own’. People who felt that their distress was in some way invalid, or that services were ‘prioritising others’, were reluctant to seek help. Previous studies have shown that self-appraisal moderates emotional sharing and seeking help from informal sources of support, such as friends and families.^45,46^ We think that our study is among the first to show that this process underlies an intrinsic reluctance to seek help from official support services. Perhaps, preventive psychosocial interventions might focus on reducing negative self-appraisals that compound experiences of distress and reluctance to seek help.

Second, given these negative tendencies, our participants reported that finally receiving validation of their suffering and their entitlement to care, particularly from someone who they perceived to have special expertise, changed the ways in which they perceived their distress and eligibility for psychosocial care, which, in turn, facilitated coping and adapting. This finding is consistent with the theoretical work of Maercker and Horn,^47^ who argue that social acknowledgement (defined as survivors’ experience of reactions from society toward their unique state and difficult situation) has an important effect on psychosocial adaptation to the primary stressor after adverse events. Authoritative validation can be viewed as a specific source and type of social acknowledgement that is a crucial component in psychosocial care.

However, people also report that being told that their experiences are ‘normal’ can be invalidating. With good intentions, it is likely that service providers were trying to convey ‘it's okay to not be okay’. However, the message that came through to our interviewees was that their distress was so minor that it was just part of the normal experience. Thus, the problem with ‘normalising’ reactions to major events is that it can minimise people's experience of distress and delegitimise their need for care. This is particularly concerning given that there is growing evidence that invalidating experiences can be more powerful than validating experiences.^45,46^ Finding that some people experienced authoritative invalidation as a secondary stressor that compounded their distress and mitigated coping, and recovery is of particular concern. This emphasises the importance of ensuring that processes of validating people's experiences by people whose opinions they respect are available. This raises an important matter for messaging and for the availability of services. It suggests that improvements to delivering the well-being and psychosocial agendas is necessary, with more focus on social and authoritative validation in services’ responses.

When distressed people did decide to seek care, some did not know how to find the right care, and thought that active outreach from the Hub should have been provided earlier. Some felt that the delays in accessing psychosocial care were not favourable to their coping and recovery, and acted as a secondary stressor. In addition, many parents felt that it was difficult to get access to early help and advice about the best ways in which they could support their children. They described how this compounded their personal distress and, if available, this kind of care might have enabled them to provide better support to their children, who were also affected. In contrast, learning that outreach services were available and easily accessible to them, if needed, helped to mitigate people's distress. The implication of these findings is that people require early and open access to authoritative sources of information and emotional support.

Many interviewees reported that it was unhelpful to recurrently encounter barriers when attempting to access more specialist care. Some people thought that the stress engendered by these barriers also exacerbated their distress and acted as a secondary stressor. People benefitted from the assistance provided by the Hub in finding care when they did not know where to go or were encountering barriers. This finding emphasises the importance of planning, care pathways and the importance of outreach. It argues for coordinating outreach and offering the expertise of authoritative practitioners employed at Hubs by specialist mental health services.

Our interviews indicate that people who are affected regard the advice of their GPs as important. Many of our interviewees visited their GPs during the early response to seek validation and advice and, in some cases, referral. This finding is consistent with recent research that shows that GPs are the primary or first healthcare provider to whom people turn in the immediate aftermath of terrorist attacks.^10,20,21^ Our interviewees described variable responses. Some encountered limited knowledge of the psychosocial aspects of major incidents and available services, and inappropriate offers of help, including medication. To our knowledge, only one previous study has investigated people's experiences of consulting GP services after a terrorist attack.^10^ This also found that most thought their GP had not been helpful or provided inappropriate care. Unhelpful experiences of primary care can have adverse consequences. It can reduce further attempts at help-seeking and, in some cases, exacerbates distress and affects coping and recovery. We think that this information confirms the vital role of GPs and the importance of briefings for them to expand their knowledge.

The findings from our interviews substantiate the importance of people's social connectedness and social support. The Hub, in partnership with other services, was instrumental in organising local support groups, family days and workshops. People who attended these events benefitted from authoritative validation, useful information and advice. Importantly, they valued the social connection with others who shared the experience. Moreover, the relationships that developed from these events became important sources of longer-term social support and enabled coping, adapting and recovery. Indeed, several interviewees called for earlier facilitation of survivors’ groups and signposting to online groups for people with shared experience. This reinforces the finding that people who are distressed need psychosocial interventions based on the principles of psychological first aid, and particularly their need for social connectedness and social support.^13,48,49^

Taken together, these findings support the view that more emphasis on psychosocial interventions is required in early outreach responses. The ubiquity of distress, the apparent limitations of psychiatric screening measures and the important role that psychosocial processes play in moderating distress and facilitating coping demonstrate the need for more attention to be given to the well-being and psychosocial agendas in future service responses to incidents.

### Strengths and limitations

Self-reports and historical and subjective accounts of early distress and experiences of social support have inherent recall bias.^50^ Hence, self-report might be a potential limitation of the study. For example, we acknowledge that depression or dysthymia at the time of interview may have played a role in shaping or colouring our participants’ accounts of their lived experiences before the incident. Second, it is possible that some survivors may have elaborated or falsified their experience of the event and the aftermath. However, our interviewers spent at least an hour with each participant, and the level of detail in their accounts and their affective responses were consistent with their accounts, conveyed conviction and were powerful. The researchers also spent considerable time reading and re-reading the transcripts of the interviews, and it appeared that the participants’ accounts of their experiences contained a level of detail that was consistent with their reported level of post-event distress on the screening. Moreover, our access to longitudinal screening data over 3 years mitigated this limitation, in that participants’ historical and subjective accounts could be cross-checked with their initial and serial screening scores. Hence, we were not persuaded that elaboration or falsification were major factors influencing the data.

There is good evidence that prior mental health problems, female gender and younger age are key pre-disaster risk factors.^51^ Having children affected by the event has also been associated with poor mental health outcomes,^43^ perhaps because of greater concern, responsibilities and stress during and after the event. Severity of exposure is also a key risk factor in people's responses post-event, often showing a dose–response relationship.^18^ This study selected a purposive sample representing the range of initial distress and recovery trajectories and exposure levels, age (young through to middle-aged adults) family status (parents and young people) and geography.^45^ We acknowledge that our sample has a gender bias in that it was predominantly female. As such, it was representative of the audience at the Arena and the research positive cohort. This gender bias could have implications for the interpretation of some of our findings.

We recognise that the subset of Hub registrants from which our sample was drawn may not be fully representative of the much larger group of 19 500 people who were in attendance at the Arena. This wider group may have had somewhat different experiences, given that they did not feel the need to use the Hub.

Another limitation is self-selection bias in the sample derived from the Hub. All participants had responded to active outreach and completed screening measures, volunteered for research and consented to recruitment to this particular study. This could have attracted more people with more severe distress. However, this inherent bias was mitigated by recruiting a purposive sample of people that included those with mild and moderate distress reactions.

The study explored participants’ subjective experiences of distress, help-seeking and psychosocial care, using qualitative methods. This is rare in the existing literature on major events. It has privileged survivors’ voices and demonstrates that qualitative approaches can provide valuable insights into people's experiences of distress and its course, which is lacking in quantitative approaches. Notwithstanding its strength, the findings are based on only a small sample and might not be representative of all survivors. A larger-scale quantitative survey study is in progress to offset this potential weakness, which we hope will reduce the risk of false interpretations.

A further limitation of this qualitative research study is that it has not been possible to pursue one of our original objectives, that is, to identify any possible underlying causal psychosocial mechanisms or processes mediating participants’ experience of distress. Our larger-scale survey study and the subsequent quantitative data analysis will address this omission.

A strength of the study is that it explored the use and experiences of a wide range of both existing and newly established post-event support services. A limitation is its specificity, in that the findings may only have relevance to relatively well-resourced care systems that have the advantage of universal healthcare coverage and an active outreach programme.

In conclusion, the study enhances the understanding of people's natural, common reactions and psychosocial processes, and experiences of services and psychosocial care following the Manchester bombing.

Our conclusion is that it is very important for all of the agencies to come together well before any incident, to agree a comprehensive plan using a tripartite framework as published by Murray et al,^52^ that provides a way of understanding and responding to the many psychosocial and mental health effects of critical incidents. These interventions fall into the categories of supporting the well-being of everyone affected (the Wellbeing Agenda); providing support and focused psychosocial interventions to meet the needs of people who are struggling or have become distressed, but who do not reach the threshold for specialist mental health assessment and treatment (the Psychosocial Agenda); and identifying, assessing and meeting the needs of a smaller number of people who may develop conditions that require specialist mental health assessment and, possibly, treatment (the Mental Health Agenda). This approach to major incidents is now included in recently published guidance for the National Health Service in England.^17^

This conclusion has several implications. It is important to be aware of the potential duration of the effects of incidents on people who are affected. Although a minority of people require mental health services, there is a much larger group of people who become distressed who do not require specialist mental healthcare but who do require psychosocial care. Provision for this group of people may not be adequate after many incidents. The authors observe that there is a substantial agenda within all services for developing awareness of people's needs after incidents, and another for training and developing practitioners.

We think that our findings have implications for policy by enabling planners to take them into account when they design services for responding to events, as well as for practitioners when incidents occur.

## Data Availability

The data from the qualitative interviews are not publicly available because they contain information that could compromise the privacy of the research participants.

## Supplementary material

For supplementary material accompanying this paper visit http://doi.org/10.1192/bjo.2022.2. click here to view supplementary material
